# Case report: JAK inhibitor treatment of immune dysregulation symptoms in a patient with PTPN2 deficiency

**DOI:** 10.3389/fimmu.2024.1523256

**Published:** 2025-01-31

**Authors:** Anna Roppelt, Ulyana Markina, Irina Beloglazova, Vasily Parshin, Dmitry Kanner, Dmitry Pershin, Mariia Fadeeva, Elena Raykina, Maxim Aleksenko, Alexander Karaulov, Mariana Lysenko, Daria Fomina

**Affiliations:** ^1^ Moscow Research and Practical Center of Allergy and Immunology, Clinical City Hospital 52, Department of Health of the City of Moscow, Moscow, Russia; ^2^ Department of Therapy, Pirogov Russian National Research Medical University, Moscow, Russia; ^3^ Moscow Oncology Hospital 62, Department of Health of the City of Moscow, Moscow, Russia; ^4^ Laboratory of Hematopoietic Stem Cell Transplantation and Immunotherapy and Laboratory of Molecular Biology, Dmitry Rogachev National Medical Research Center of Pediatric Hematology, Oncology and Immunology, Moscow, Russia; ^5^ Department of Clinical Immunology and Allergy, First Sechenov Moscow State Medical University (Sechenov University), Moscow, Russia; ^6^ Department of Pulmonology, Astana Medical University, Astana, Kazakhstan

**Keywords:** PTPN2, immune dysregulation, inborn errors of immunity, JAK-inhibitor, case report

## Abstract

A heterozygous mutation in the *PTPN2* gene has recently been described in several patients exhibiting symptoms of immune dysregulation. The gene encodes a ubiquitous non-receptor T-cell protein tyrosine phosphatase that exerts a negative feedback on the JAK–STAT pathway. Limited clinical data are available advocating the use of JAK inhibitors as an effective treatment for autoimmune complications of PTPN2 deficiency. However, the mechanism of pathogenesis for these complications suggests this possibility. We report on a 32-year-old male patient with interstitial lung disease, cytopenia, and lymphadenopathy accompanied by *de-novo* deletion in *PTPN2*. The patient has been receiving systemic steroid treatment for decades, which has resulted in hormone dependence as well as therapy-related adverse side effects. After the diagnosis of PTPN2 deficiency, treatment with the JAK inhibitor ruxolitinib was initiated at a dose of 15 mg per day, which was escalated to 30 mg daily after 1 month. The steroid treatment was discontinued within 3 months. At the 9- and 16-month checkpoint, after 6 and 13 months correspondingly of monotherapy with ruxolitinib at a dosage of 30 mg per day, the patient had stable blood counts, lymphadenopathy decreased, and the lung interstitial disease improved. Thus, according to our experience, JAK inhibitors are able to alleviate the PTPN2 deficiency symptoms, including hematological changes and interstitial lung damage.

## 1 Introduction

A heterozygous mutation in the *PTPN2* gene was recently described in several patients with immune dysregulation symptoms (1–3). Thaventhiran J.E.D. et al. reported a case study of a family, in which both the mother and her son had a heterozygous truncating variant p.Glu291* of the *PTPN2* gene. The son was diagnosed with common variable immunodeficiency at the age of 20. The manifestations were B-cell lymphopenia, low levels of immunoglobulin G, polyarthropathy, recurrent bacterial infections, splenomegaly, and inflammatory lung disease. The mother had systemic lupus erythematosus, diabetes mellitus, hypothyroidism, and autoimmune neutropenia (1). Parlato M. et al. presented a case study of a 3-year-old girl who had been suffering from intestinal inflammation and eczema since the age of 3 months. The heterozygous missense variant p.Cys216Gly in exon 6 of the *PTPN2* gene was identified in this individual via whole exome sequencing (2). Jeanpierre M. et al. identified heterozygous variants in *PTPN2* in six patients with systemic autoimmunity such as lupus erythematosus and Evans syndrome (3).

The *PTPN2* gene encodes a ubiquitous non-receptor T-cell protein tyrosine phosphatase that exerts a negative feedback on the JAK–STAT pathway (4). Thaventhiran J.E.D. et al. demonstrated IFNγ-induced hyperphosphorylation of STAT1 in T-cell blasts of PTPN2-deficient patients (1). Meanwhile, Parlato M. et al. showed increased phosphorylation of STAT1 and STAT3 in response to IL-21 in a patient’s EBV-B cells compared to a healthy control (2).

Based on *in-vitro* studies, JAK inhibitors are proposed to be an effective treatment for autoimmune complications of PTPN2 deficiency (2, 3). However, to our knowledge, there are currently no data on the use of JAK inhibitors in patients with *PTPN2* deficiency.

Here, we report a case of an adult male patient with interstitial lung disease, cytopenia, and lymphadenopathy accompanied by *de-novo* deletion in the *PTPN2* gene. We also discuss our experience with his treatment with the JAK inhibitor ruxolitinib and the treatment scheme choice.

## 2 Case report

We presented a case of a 32-year-old Caucasian male patient with features of immune dysregulation. At the age of 1.5 years, the patient had lymphadenopathy in different lymph node groups, which was initially diagnosed as infection-associated lymphadenitis. At 16 years old, he developed hemolytic anemia, which improved after receiving intravenous immunoglobulin and steroids. After steroid discontinuation, the patient developed pneumonia, hepatosplenomegaly, generalized lymphadenopathy, and thrombocytopenia (platelet count of 38 × 10^9^/L). Bone marrow aspiration results and lymph node biopsy excluded hemoblastosis and indicated reactive lymphadenopathy. The patient’s durable lymphopenia and hypogammaglobulinemia raised suspicion of an inborn error of immunity (IEI), and he was referred to immunologists. The target genetic screening for mutations in *SН2D1А* and *BIRC4* genes was negative. The patient was diagnosed with common variable immunodeficiency and further treated with a complex therapeutic algorithm including intravenous immunoglobulin replacement, antibiotics, and antifungal drugs. Immunological investigations at diagnosis are demonstrated in **Table 1**. The treatment resulted in partial efficacy in terms of lung foci. In order to treat thrombocytopenia, steroid pulse therapy was administered. As a basic immunosuppressant, azathioprine was started but soon discontinued due to treatment-associated agranulocytosis.

**Table 1:** Immunological investigations at the diagnosis of CVID (16 years old), before ruxolitinib treatment (30 years old), and 16 months after ruxolitinib initiation (32 years old).

Age (years)	Lymphocytes (10^9^/L)	CD3^+^CD4^+^ (10^9^/L)	CD3^+^CD8^+^ (10^9^/L)	CD3^−^CD19^+^ (10^9^/L)	CD3^−^CD56^+^CD16^+^ (10^9^/L)	CD4/CD8	IgG (g/L)	IgM (g/L)	IgA (g/L)
16	1.71	0.14	1.25	0.05	0.17	0.11	3.13	0.28	0.15
30	1.0	0.42	0.54	0.02	0.07	0.78	8.26^*^	0.79	0.05
32	0.61	0.18	0.3	0.01	0.09	0.6	8.5^*^		

* Trough IgG on IVIG replacement therapy 0.6 g/kg/month.

**Figure 1:**
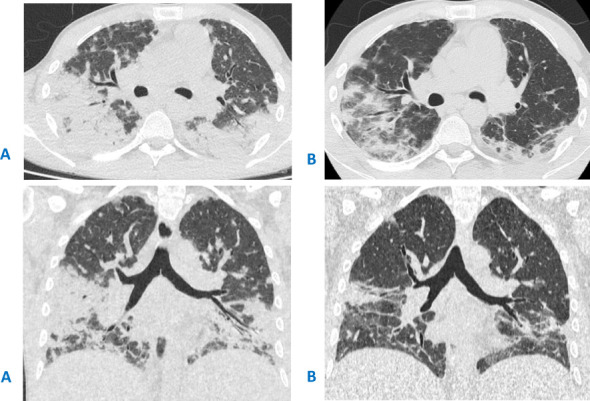
**(A)** CT scan before the thoracoscopic lung biopsy and steroid initiation, showing massive bilateral areas of airless lung tissue, more prominent in the lower lobes, located in both central and peripheral areas. **(B)** CT scan after 2 weeks of treatment with high doses of steroids.

The patient continued to receive steroids, but each attempt to taper the medication decreased the platelet count and caused new foci in the lungs. At the age of 27, respiratory failure occurred after another attempt at steroid cancelation, accompanied by subtotal lung damage observed on CT scans (**Figure 1A**). Despite antibiotic and antifungal (amphotericin) therapy, no improvement was observed. Thoracoscopic lung biopsy revealed cryptogenic organizing pneumonia. Optimal response was observed on high-steroid doses (**Figure 1B**) with slow de-escalation to low-dose regimen. However, as before, steroid tapering resulted in the recurrence of thrombocytopenia and lung lesions.

By the age of 30, the patient developed steroid hormone dependence with clinical and laboratory manifestations and also presented with Cushing’s syndrome as an adverse effect. Moreover, CMV infection (4,000 copies/mL of CMV in bronchoalveolar lavage) was detected.

**Figure 2:**
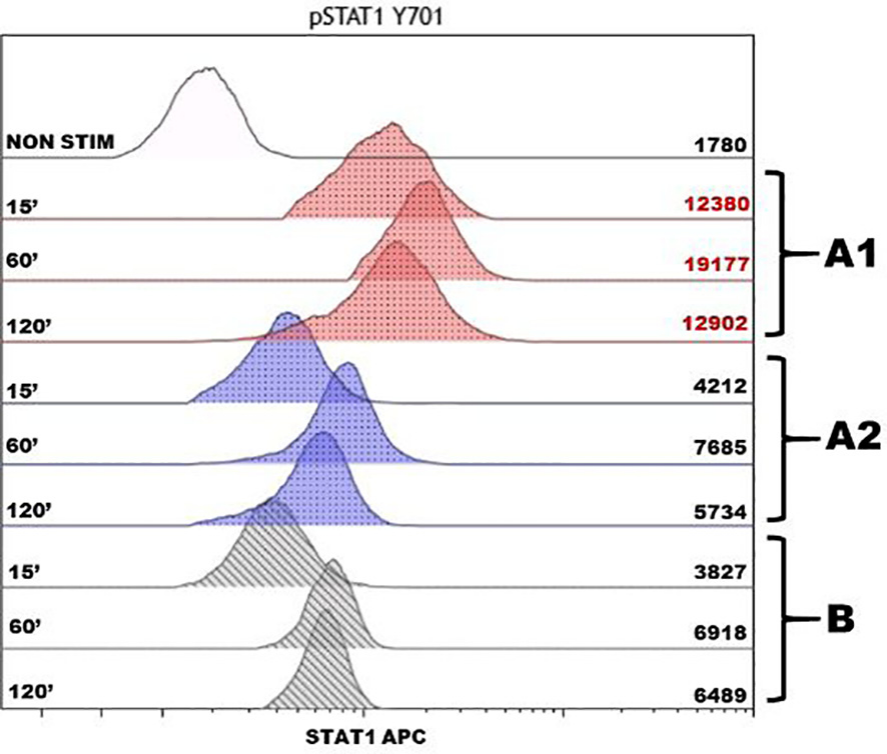
Assessment of phosphorylation of intracellular protein STAT1 after stimulation by IFNγ in monocytes of the patient and healthy donor. The histogram displays the analysis of the STAT1 protein phosphorylation, with the stimulation time in minutes (15, 60, and 120 min) on the left and the mean fluorescence intensity on the right. The non-stimulated sample is noted as NON STIM, the red color shows the phosphorylation points of the patient before the therapy **(A1)**, the blue color shows after 1 year of the JAK inhibitor therapy course **(A2)**, and the gray color shows a control sample from a conditionally healthy person **(B)**. To determine the kinetics of phosphorylation, we compared the fluorescence intensity of the samples stimulated during different time intervals with an unstimulated sample. The in-house references showed the enhanced phosphorylation of the STAT1 protein in the patient before therapy with a maximum value of 60 min. After the course of therapy, the result complied with the reference values and was comparable with the protein phosphorylation in a conditionally healthy person.

For further patient diagnosis, target panel sequencing of IEI-associated genes was performed. The sequencing detected heterozygous deletion of exons 1 and 2 in the *PTPN2* gene. This variant has never been previously described in the literature. MLPA analysis confirmed the mutation; the examination of generally healthy parents showed its *de-novo* status. The literature suggests that the *PTPN2* product inhibits the JAK–STAT pathway. Phosphorylation assays of STAT1 and STAT3 demonstrated hyperphosphorylation in patients with heterozygous mutations in *PTPN2* (1, 2). In our case, the STAT1 phosphorylation assay also demonstrated hyperactivation of the STAT1 pathway (**Figure 2**).

**Figure 3:**
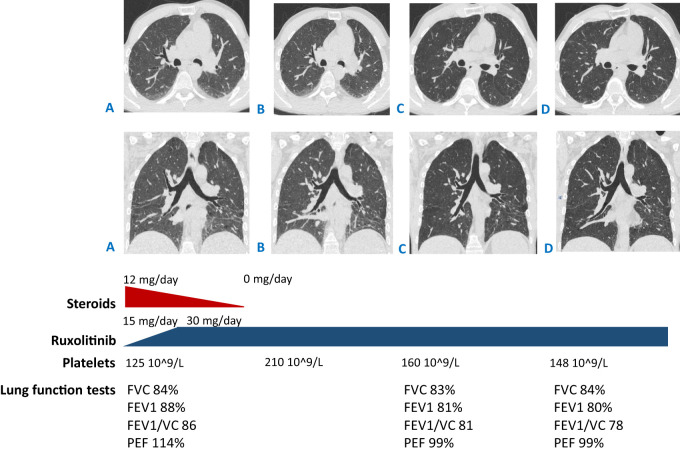
CT scans: **(A)** at the beginning of ruxolitinib treatment (started from the dose of 15 mg/day then escalated to 30 mg/day) and steroid tapering (from the dose of 12 mg/day of methylprednisolone to complete withdrawal); **(B)** after 3 months of ruxolitinib treatment and straight after complete steroid discontinuation; **(C, D)** 9 and 16 months since ruxolitinib initiation (which is 6 and 13 months of monotherapy with ruxolitinib correspondingly, 30 mg per day). **(A, B)** Reduced pneumatization of the lung tissue in the basal sections, with no dynamics in the areas of hypostatic changes between **(C, D)** improved pneumatization in the basal sections, with less prominent reticular changes. Shown below are the lung function tests in time correlation with CT scan performance. FVC, forced vital capacity; FEV1, forced expiratory volume in 1 s; VC, vital capacity; PEF, peak expiratory flow.

Considering the genetic results and the adverse side effects of steroid therapy, the immunosuppressive treatment was adjusted: the JAK inhibitor ruxolitinib was initiated at a dose of 15 mg per day. The dose was escalated to the optimal one according to the label—30 mg per day—after a month. Finally, the steroids were completely canceled after 3 months (**Figures 3A, B**).

The therapy was well-tolerated. Due to the development of mild CMV viremia (1,100 copies/mL) during the ruxolitinib therapy, valganciclovir was started at a 5-month period after JAK inhibitor initiation. In the 2-month treatment, no copy of CMV was detected and valganciclovir was continued in a prophylactic dose until 16 months of checkpoint when the therapy was completely canceled. Throughout this period, no virus was detected in the blood. The last control of CMV viremia was 6 months after valganciclovir cancellation and it still was negative. The patient had no signs of organ damage and no other herpes virus infection has been reported since JAK inhibitor initiation.

At the 3-month checkpoint, the patient had stable blood counts with a tendency toward an increase in platelets. A positive response was observed with CT scan visualization. After 9 months (6 months of monotherapy with ruxolitinib 30 mg per day), stable blood counts and decreased lymphadenopathy were observed. The patient gained 7 kg; all the foci previously presented on the CT scan vanished and no new foci appeared. Pneumatization in the basal sections of the lungs was improved and reticular changes became less prominent (**Figure 3C**). At the 16-month checkpoint, all the symptoms were still under control (**Figure 3D**). It is worth noting that the patient was still lymphopenic; therefore, he continued to receive prophylactic co-trimoxazole along with IVIG replacement therapy (**Table 1**). Nevertheless, evaluation of STAT1 phosphorylation a year after JAK inhibitor initiation showed results comparable with protein phosphorylation in a conditionally healthy person (**Figure 2**). In addition, the patient’s social adaptation has benefited from the treatment: he has noted an improvement in his general wellbeing and has adopted a more positive outlook on his future. According to the SF-36 Health Survey conducted before and 16 months after ruxolitinib initiation, the patient’s physical health score increased from 41.6 to 53.4 and his mental health score increased from 19.6 to 28.6 correspondingly. The numbers reflect the improvement of the patient’s quality of life.

## 3 Discussion

The JAK–STAT pathway serves as a crucial checkpoint for several IEIs, including STAT1 and STAT3 gain-of-function IEIs. PTPN2 deficiency appears to be a newly discovered disease resulting from compromised STAT-mediated signaling leading to its hyperactivation.

The findings from a cohort of patients with STAT1 and STAT3 GOF mutations demonstrated the long-term treatment efficacy of ruxolitinib or tofacitinib in symptoms of immune dysregulation (5). Single cases additionally showed clinical improvement of interstitial lung disease in patients with STAT3 GOF disease treated with ruxolitinib or tofacitinib (6, 7).

Parlato M. et al. performed *in-vitro* assays confirming the inhibition of STAT1 and STAT3 phosphorylation upon ruxolitinib addition to PTPN2-deficient patients’ cells (2). Jeanpierre M. et al. showed the inhibitory effect of tofacitinib on cytokine-induced T-cell proliferation using samples from patients with PTPN2 haploinsufficiency (3). These experiments provided a solid background to forecast the efficacy of JAK inhibitors toward immune dysregulation in patients with PTPN2 deficiency, along with the resolution of autoimmune features upon JAK inhibitor therapy in patients with STAT1 and STAT3 GOF mutations.

Simoncic P.D. et al. indicated that JAK1 and JAK3 are physiological substrates of T-cell protein tyrosine phosphatase (4). Nevertheless, the scarcity of publications and the absence of clinical examples make the choice of the preferable JAK inhibitor treatment unclear. Ruxolitinib is a selective inhibitor of JAK1 and JAK2 (8), and tofacitinib blocks JAK1, JAK2, JAK3, and, to a lesser extent, TYK2 (9).

The here-reported mutation in *PTPN2* has never been previously described in the literature. Deletion of exons 1 and 2 affects the PTP catalytic domain, which in turn probably disrupts the catalytic activity of the enzyme and compromises the dephosphorylation of STAT peptides (1, 2). The assumption is consistent with the hyperactivation of the STAT1 pathway observed in our patient before the JAK inhibitor therapy.

The patient was treated with ruxolitinib as a more selective JAK inhibitor. The therapy was well-tolerated; however, one must be wary of CMV infection. It resulted in a rapid and significant improvement in thrombocytopenia and overall condition, allowing the withdrawal of steroids. There was also an improvement in interstitial lung disease although slow. It is unclear whether a more rapid effect could be achieved with an alternative JAK inhibitor or higher doses of ruxolitinib. It is likely that JAK inhibitor therapy mediates the delayed response of interstitial lung disease in PTPN2-deficient patients. Further analysis of larger patient groups is necessary to address these issues.

It is worth noting that STAT1 phosphorylation in the described patient decreased after the course of ruxolitinib and complied with the reference values. The results of a one-time measurement before and during therapy should be taken with caution. Repeated measurements in the same patient in order to obtain an average value, as well as an evaluation of STAT1 phosphorylation in a cohort of patients with PTPN2 deficiency, are required. However, the experience of each individual case is important in terms of the limited information about this disease up to date. If relevant results are obtained in a large group of patients, measurement of STAT1 phosphorylation activity can be used to assess the response to the JAK inhibitor treatment.

In conclusion, patients with РТРN2 deficiency exhibit features of IEI with immune dysregulation. According to our experience, JAK inhibitors are able to alleviate hematological and interstitial lung disease symptoms. To our knowledge, this is the first publication reporting the use of JAK inhibitors in patients with PTPN2 deficiency and immune complications. The rarity of this disease makes the description of each individual case of great value.

## Data Availability

Datasets are available on request: The raw data supporting the conclusions of this article will be made available by the authors, without undue reservation.
